# Heterogeneity of the NIH3T3 Fibroblast Cell Line

**DOI:** 10.3390/cells11172677

**Published:** 2022-08-28

**Authors:** Amir Mohammad Rahimi, Mingfang Cai, Sigrid Hoyer-Fender

**Affiliations:** Johann-Friedrich-Blumenbach-Institute of Zoology and Anthropology—Developmental Biology, Göttingen Center for Molecular Biosciences (GZMB), Ernst-Caspari-Haus, Georg-August-Universität Göttingen, Justus-von-Liebig-Weg 11, 37077 Göttingen, Germany

**Keywords:** NIH3T3, fibroblasts, myofibroblasts, proliferation, primary cilia

## Abstract

The embryonic mouse fibroblast cell line NIH3T3 is widely used in life science research, including the study of cell cycle control and primary cilia. Fibroblasts are the most important cell type in connective tissue, as they produce components of the extracellular matrix and determine tissue architecture. However, they are very heterogeneous and consist of subtypes specific to their organ of residence, among others. The NIH3T3 cell line was derived from whole mouse embryos that developed to pre-birth and is therefore most likely composed of different fibroblast subtypes. Furthermore, prolonged proliferation may have influenced their cellular composition. A heterogeneous cell population is unsuitable for any sophisticated research project. We found that the proportion of ciliated cells in the total NIH3T3 cell population was highly variable and asked whether this was a consequence of cellular heterogeneity and what molecular signatures were associated with it. We have established sub-cell lines by clonal expansion of single cells and characterized them morphologically and molecularly. Eventually, a myofibroblast-like and a fibroblast-like cell line were generated that differ in ciliation and proliferation. These homogeneous cell lines are valuable for a more detailed study of their molecular signatures, not least to uncover further the molecular pathways that contribute to the formation of the primary cilium.

## 1. Introduction

Fibroblasts are mesenchymal cells of connective tissue. They produce extracellular matrix components, primarily collagens type I and III, and thus define tissue architecture by supporting the topography of organs. Furthermore, they are crucially involved in wound healing and drive inflammation and scarring [1]. Collectively, fibroblasts are multifaceted players in health and disease driving, i.a., fibrosis, arthritis, and cancer. They perform lineage-specific functions, as well as specialized functions required by their organ of residence. Fibroblasts are indeed heterogeneous cells, and even within one tissue, diverse subtypes of fibroblasts exist, as has been revealed by single-cell RNA sequencing [2,3,4]. Their transcriptional profiles indicated the existence of universal and specialized subtypes and led to the suggestion that universal fibroblasts can differentiate into specialized fibroblasts and thus provide the resource cell pool for the functional heterogeneity of fibroblasts. Universal and specialized subtypes, as well as activated fibroblasts in the perturbed disease state, exist altogether in the same tissue.

Fibroblasts also play a major part in wound healing. The wound healing process comprises three phases: the inflammatory, the proliferative, and the regenerative phases, in which fibroblasts become activated and transform into myofibroblasts [5]. Myofibroblasts are highly contractile cell types and are classically characterized by their expression of alpha-smooth-muscle-actin (α-SMA) but also show upregulation of fibronectin and collagen and the formation of stress fibers [6,7]. In addition, myofibroblasts have extensive rough ER and are larger than fibroblasts [8]. The differentiation into myofibroblasts is activated by mechanical stress, and TGF-β is the major signaling pathway [9]. The TGF-β pathway signals through the primary cilium. Upon ligand binding, the activated receptors are translocated from the ciliary tip toward the base of the cilium, where SMAD transcription factors are activated [10]. The primary cilium, therefore, plays a key role in the formation of myofibroblasts, first through the uptake of TGF-β1 receptors and second through the regulated process of cilium assembly and disassembly [1,11]. That is, myofibroblastic transformation is manifested by the initial growth of a primary cilium, which is crucial for the progression of trans-differentiation, followed by the complete loss of the primary cilium when the myofibroblastic phenotype is reached [1]. Furthermore, deciliation essentially depends on the transition to the myofibroblast state [1].

Primary cilia are microtubule-based organelles anchored to the cell membrane with the basal body that protrude into the environment [12,13,14,15]. Almost all cells of the body harbor a single or primary cilium. Primary cilia are important signaling hubs that transmit mechanical and chemical cues to the cell interior [16,17]. They are fundamental for crucial cellular functions, such as proliferation and differentiation, and thus important for development and tissue homeostasis, leading to severe diseases when disrupted [18,19].

The presence of primary cilia on cultured fibroblast cells has long been known, as it plays an essential role in determining the direction of cell migration to the wound and in wound closure [20,21,22,23,24]. The mouse fibroblast cell line NIH3T3 is a prevalent model system for studying primary cilia. The NIH3T3 cell line was originally derived from a 17- to 19-day-old whole Swiss mouse embryo [25]. NIH3T3 cells are easy to maintain in culture and are therefore suitable for a wide range of applications in cell and molecular research. Furthermore, primary cilia are easy to detect by immunocytology using antibodies against the marker proteins acetylated α-tubulin and/or ARL13b [26,27,28]. The formation of primary cilia depends on the cell cycle, and they are mainly found in quiescent cells [20,29,30,31,32]. Culturing NIH3T3 cells in a medium with a low serum concentration, a so-called serum-deprived or serum starvation medium, resulted in cell cycle arrest accompanied by the formation of primary cilia, a method generally used to induce primary cilia. We commonly observed that the percentage of ciliated NIH3T3 cells is quite variable even when primary cilia formation was induced by serum deprivation, and that a large fraction of NIH3T3 cells is not ciliated even under non-proliferating conditions. Different abilities to form a cilium indicate heterogeneity in the cell population and raise the question of whether there are molecular signatures that promote or enable the formation of a primary cilium. To decipher the specific properties of cells that form a cilium, we first generated sub-cell lines from single cells and then focused on two sublines that differed in their ability to generate primary cilia. Morphological and molecular analyses finally showed that two distinct sublines, F2 and C11, differed in proliferation and ciliation. Their different proliferation rates are maintained for at least eight months of permanent cultivation. Establishing diverse clonal sublines of NIH3T3 cells allows for a closer examination of their molecular signatures to further uncover the molecular pathways that compel the cell to form a primary cilium.

## 2. Materials and Methods

*Cell cultivation and clonal expansion of single cells*

NIH3T3 cells were obtained from DSMZ (Deutsche Sammlung von Mikroorganismen und Zellkulturen, Braunschweig, Germany; ACC59), and thereafter named ‘DSMZ’ cells. Cells were grown and maintained in Dulbecco’s Modified Eagle’s Medium (DMEM; GlutaMax^TM^ with high glucose concentration (4.5g/L); ThermoFisher Scientific, Waltham, MA, USA, #10566), supplemented with 10% (*v*/*v*) fetal calf serum (FCS; referred to as the standard or the normal medium (NM)) or 0.5% (*v*/*v*) FCS for the serum starvation medium (SSM), and 1% penicillin/streptomycin (1000 U/mL penicillin and 1000 µg/mL streptomycin) at 37 °C and 5% CO_2_ [33]. Cells were regularly passaged into new flasks upon being approximately 80% confluent. Based on the experiment, 96-, 24-, 12-, or 6-well plates (ThermoFisher Scientific), or 25- and 75-cm^2^ cell culture flasks (TPP Techno Plastic Products AG) were used. For the clonal expansion of single cells, the cells were trypsinized, and the cell suspension was diluted according to a calculated number of one cell per 100 µL medium and sub-cultured in 96-well plates. Cell growth was regularly inspected microscopically, and sub-cell lines originating from a single cell were chosen for clonal expansion.

*Immunocytology*

Cells were grown on glass coverslips in 6-well plates using an initial number of 2.5 × 10^5^ cells per well. Cells were then fixed in 3.7% paraformaldehyde (PFA) for 20 min at 4 °C and permeabilized with 0.3% Triton X-100 in PBS (phosphate-buffered saline) for 10 min at room temperature. After rinsing in PBS, non-specific binding sites were blocked by incubation in PBS containing 1% bovine serum albumin (BSA) and 0.5% Tween-20 for at least 1 h. Samples were incubated with the primary antibodies anti-acetylated α-tubulin (clone 6-11B-1; Santa Cruz Biotechnology, Inc.; #sc-23950, diluted 1:100), anti-ARL13b (Proteintech, Germany, St. Leon-Rot, Germany; #17711-1-AP, diluted 1:400), anti-vimentin (Santa Cruz Biotechnology, Inc.; #sc-7557, diluted 1:50), or anti-alpha-smooth muscle actin (clone 1A4, eBioscience^TM^, Invitrogen; #14-9760-80) and with Phalloidin-TRITC (Phalloidin-tetramethylrhodamine B-isothiocyanate, Merck, Darmstadt, Germany; #P1951) at 4 °C overnight. Secondary antibodies used were goat-anti-mouse-IgG-DyLight 488 (#35503, ThermoScientific, Waltham, MA, USA; diluted 1:300), goat-anti-mouse-IgG-AlexaFluor555 (#A21422, Lot 948498, ThermoScientific, Waltham, MA, USA), and goat-anti-rabbit-IgG-MFP590 (#MFP-A1037, Mobitec, Göttingen, Germany; diluted 1: 100). DNA was counterstained with DAPI (4′,6-diamidino-2-phenylindole; Vector Lab., cat. no. H-1500). For the induction of primary cilia, serum concentration was reduced to 0.5%, and cells were incubated in the serum-starved medium for 48hrs before immunocytology. Images were taken by confocal microscopy (LSM 780, Carl Zeiss AG, Oberkochen, Germany) and processed using FIJI and Adobe Photoshop 7.0. Primary cilia were visually inspected through different focal planes and manually counted. For each replicate, approximately 500 cells were counted. Nuclear sizes were measured with ZenBlue software (Carl Zeiss, Oberkochen, Germany).

*Immunoblotting*

24 h after seeding, cells were trypsinized, counted, and washed three times with PBS. Cells were either lysed in 1× SDS-sample buffer at 95–100 °C for 5 min or incubated first in RIPA buffer (containing 1% NP-40, 1% Triton X-100, protease inhibitors) for 15 min followed by protein denaturation in SDS-sample buffer. Protein lysates were separated on denaturing SDS-polyacrylamide gels [34] and transferred onto a Hybond ECL membrane (Amersham Hybond-ECL, GE Healthcare, Chicago, IL, USA) [35]. Membranes were blocked in TBST (Tris-buffered saline) containing 5% dry milk and 0.5% Tween-20 for at least 1 h at room temperature, followed by incubation with the primary antibodies mouse anti-acetylated α-tubulin (clone 6-11B-1, #sc-23950; Santa Cruz Biotechnology, Inc., Santa Cruz, CA, USA) or anti-alpha-smooth muscle actin (clone 1A4, eBioscience^TM^, Invitrogen; #14-9760-80) and rabbit anti-ß-actin (#ab8227, Abcam, Cambridge, UK) at 4 °C overnight in roller flasks. The primary antibodies were detected by the fluorescent-labeled secondary antibodies goat-anti-mouse-IgG-IRDye800CW (#925-32210, LI-COR Biosciences GmbH, Bad Homburg, Germany) and goat-anti-rabbit-IgG- IRDye680RD (#925-68071, LICOR Biosciences GmbH, Bad Homburg, Germany). Images were captured using LICOR Odyssey CLx (LI-COR Biosciences GmbH, Bad Homburg, Germany), and protein bands were quantified using ImageStudio Lite (LI-COR Biosciences GmbH, Bad Homburg, Germany). The amount of ß-actin in each lane was used as the reference to calculate the relative amount of acetylated α-tubulin in each lane. Data were analysed and presented using Excel.

*Quantitative RT-PCR (qRT-PCR)*

Cells were cultivated in the standard medium for 24 h, followed by total RNA extraction using peqGOLD RNApure™ (PeqLab, Erlangen, Germany) according to the manufacturer’s instructions. Total RNA was treated with Ambion^®^ TURBO DNA-free™ DNase (Life Technologies, Carlsbad, CA, USA; #AM2238), and the absence of genomic DNA was validated by PCR using amplification of *Gapdh* or *Hprt*. cDNA synthesis was performed using a Maxima First Strand cDNA Synthesis Kit (ThermoFisher Scientific, #K1641).

Quantitative real-time PCR (qRT-PCR) was performed on a CFX96TM Real-Time System (Bio-Rad Laboratories, Inc., Hercules, CA, USA) using either EvaGreen (Solis BioDyne, Tartu, Estonia) or BlazeTaq SYBR Green qPCR mix 2.0 (GeneCopoeia, Rockville, MD, USA). Primer efficiency was validated for all primer pairs by analyzing the slope and Pearson’s correlation coefficient, and the specificity of the amplification reaction was verified by melting curve analyses. The following primers were used for expression analyses: *Hprt* (mHPRT-for2 ggagtcctgttgatgttgcc/mHPRT-rev2 gggacgcagcaactgacatt), *Gapdh* (mGapdhf CACCACCAACTGCTTAGCC/mGapdhr CGGATACATTGGGGGTAGG), *c-myc* (c-myc F TGTACCTCGTCCGATTCCACG/c-myc R TGCGGAGGTTTGCTGTGGC), and *cyclinD1* (cyclinD1F TGCCAGAGGCGGATGAGAAC/cyclinD1R GGCAGTCCGGGTCACACTTG). Three technical replicates were used for each analysis. The relative expression in each probe was calculated by ΔCt using the average Ct values of both housekeeping genes as a reference. Fold changes were calculated by relation to the average relative expression in the ‘DSMZ’ cells (ΔΔCt).

*Cell Cycle (FACS) Assay*

The sub-lines were freshly seeded into 5 mL flasks at a density of either 2.5 × 10^5^ cells and cultivated for three days in the standard medium or 4 × 10^5^ cells and cultivated for 24 h. Single cell suspensions were prepared by digestion with trypsin, and the cells were harvested by centrifugation at 300× *g* for 5 min. Cells were resuspended in PBS and fixed in ice-cold 70% ethanol while gently vortexing following the instructions of the manufacturer (Muse^®^ Cell Analyzer, Millipore Corporation, Hayward, CA, USA). Fixed cells were stored at −20 °C for at least three hours. For the FACS assay, fixed cells were centrifuged at 300× *g* for 5 min, and washed once with 1× PBS. Two-hundred microliters of the Muse^®^ Cell Cycle Reagent (Millipore Corporation, Hayward, CA, USA) was added, and cells were suspended accordingly, followed by incubation for 30 min at room temperature in the dark. The Muse^®^ Cell Analyzer was used to analyze cell cycle phases in stained cells according to the manufacturer’s instructions.

*Proliferation Assay*

Cells were initially seeded into 6-well plates using 10^4^ cells per well and cultivated in the standard medium with a two-day interval for medium exchange. Cells in triplicate wells were fixed in 3.7% PFA at daily intervals over 6 days, and nuclei were stained with DAPI (4′,6-diamidino-2-phenylindole; Vector Lab., cat. no. H-1500). Using the Zeiss Observer Z1 microscope, 20 pictures were taken from each well at random positions, and the cells were counted automatically using Image-Pro Plus 7.0 (Media Cybernetics, Inc., Bethesda, MD, USA). Since each experiment was performed in triplicate, a total of 60 pictures were taken for each time point. Cell proliferation was calculated using the binary logarithm (log2) of the average cell numbers at each time point. The doubling time in hours was calculated using the cell counts obtained between the first day after seeding (time point 0) and 72 h later, when cells are in their log-phase. The doubling time in hours was calculated as 1/slope where the slope was calculated from the regression line drawn through the binary logarithm of the average cell counts.

*Statistical analyses*

The data were processed and analyzed using Excel. The box in the boxplots represents the 25–75th percentile. The median is given as a line, the mean by a cross. The whiskers show the minimum and maximum values inside the range given by Q1 − 1.5× interquartile range (IQR) and Q3 + 1.5×IQR. Data were analyzed by Student’s *t*-test, two-tailed, homoscedastic. *p* < 0.05 *, *p* < 0.01 **, *p* < 0.001 ***, *p* < 0.0001 ****.

## 3. Results

### 3.1. Cellular Heterogeneity in the NIH3T3 Cell Line

**Figure 1:**
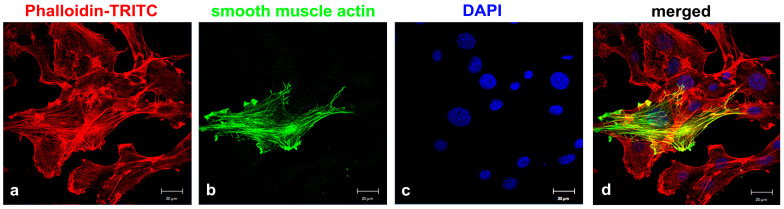
Heterogeneity of the NIH3T3 cell line (obtained from DSMZ). Cells were stained for actin with Phalloidin-TRITC (**a**) (red) and α-smooth muscle actin (**b**) (green), showing distinct expression of α-smooth muscle actin. Nuclear stain with DAPI (**c**) (blue) and merged images (**d**). Bars: 20 µm.

The established mouse embryonic fibroblast cell line NIH3T3 is a prevalent model system for studying primary cilia, and primary cilia are easily induced by cultivation in serum-deprived medium. However, we have observed that the percentage of ciliated cells varies widely and that not all cells develop a primary cilium, even when the formation of primary cilia was induced by serum deprivation under non-proliferative conditions. To omit any long-term-cultivation effects that might have influenced the cellular signatures [28], we used freshly obtained NIH3T3 cells from DSMZ (named here in short ‘DSMZ’). We found that approximately 25% of proliferating NIH3T3 cells were ciliated when cultivated in standard medium and that ciliation increased to approximately 50–75% when cells were cultivated in serum-starved medium for 24–48 h, respectively. Primary cilia were identified by staining for ciliary markers ARL13b and acetylated α-tubulin. We therefore questioned whether the NIH3T3 cell line is inhomogeneous, possibly consisting of different cell types with unequal capacities for primary cilia formation. The notion of cellular heterogeneity was confirmed by cytological detection of α-smooth muscle actin (SMA), which showed that SMA was almost undetectable in most cells, but a few cells showed strong expression of SMA (Figure 1). Thus, we intended to establish sub-cell lines from single-cell colonies to further investigate their molecular signatures and their capacities to generate primary cilia. Cells were cultivated under standard conditions, and a diluted single cell suspension was prepared that contained ~1 cell per 100 µL medium. One-hundred microliter aliquots of the cell suspension were cultivated further in 96-well plates to obtain sub-cell lines from individual cells. Wells were regularly inspected for growth of single-cell colonies, and several single-cell colonies propagated further. Finally, we obtained several sub-cell lines from individual cells and investigated their respective signatures, such as morphologies, capacities for primary cilia formation, expression of myofibroblast markers, and proliferation rates over several months.

### 3.2. Myofibroblast versus Fibroblast Cell Lines

**Figure 2:**
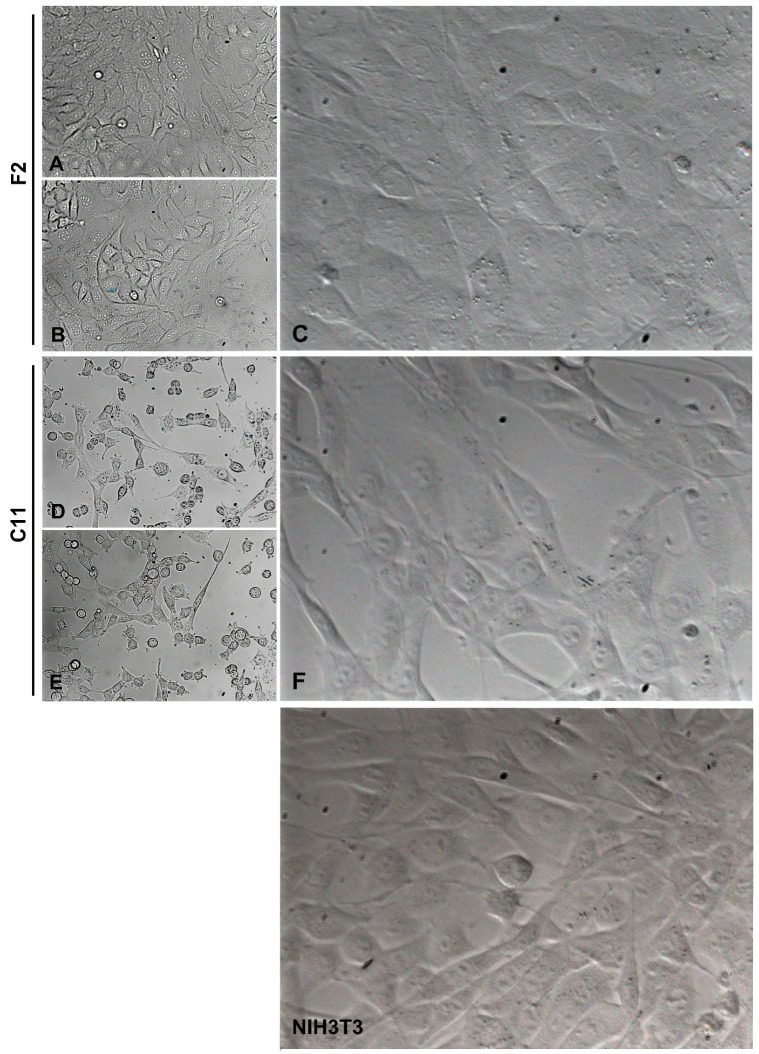
Different morphologies of sub-cell lines F2 and C11 derived from the NIH3T3 whole-cell population. F2 cells represented cubic-shaped fibroblasts, and C11 cells represented fiber-shaped fibroblasts. For comparison, the morphologies in the original NIH3T3 cell population are shown (NIH3T3). (**A**,**B**,**D**,**E**) are taken under 10× lens magnification, (**C**,**F**), and NIH3T3 are taken under 20× lens magnification. The cells were cultured in a standard medium containing 10% FCS.

We instantly observed morphological differences between sub-cell lines, i.e., two different phenotypes were identified. On the one hand, the cells showed a typical fibroblast-like morphology with a thin and elongated shape, which was subsequently named ‘spindle-shaped fibroblasts’. On the other hand, the cells were cube-shaped and appeared somewhat larger and more extended on the surface of the cell culture vessel, which was later referred to as ‘cube-shaped fibroblasts’. Representative of the typical fibroblast-like morphology or the cuboidal, rather epithelial-like morphology, are the sub-cell lines NIH3T3-C11 (in short C11) and NIH3T3-F2 (in short F2), respectively (Figure 2). Whereas F2 and C11 cells maintained their different morphologies for at least eight months of permanent cultivation, all other sub-cell lines, although established from single-cell colonies and initially allocated to either one of the two phenotypes, later on during continued cultivation, developed into both morphologies and were thus usually a mixture of spindle- and cube-shaped cells. Contamination of the original cell population could be largely ruled out, as the cells were originally authenticated as mouse cell lines by genotyping performed by the company and repeated in our laboratory, and staining for the intermediate filament protein vimentin indicated that they were indeed mouse mesodermal fibroblasts (not shown).

**Figure 3:**
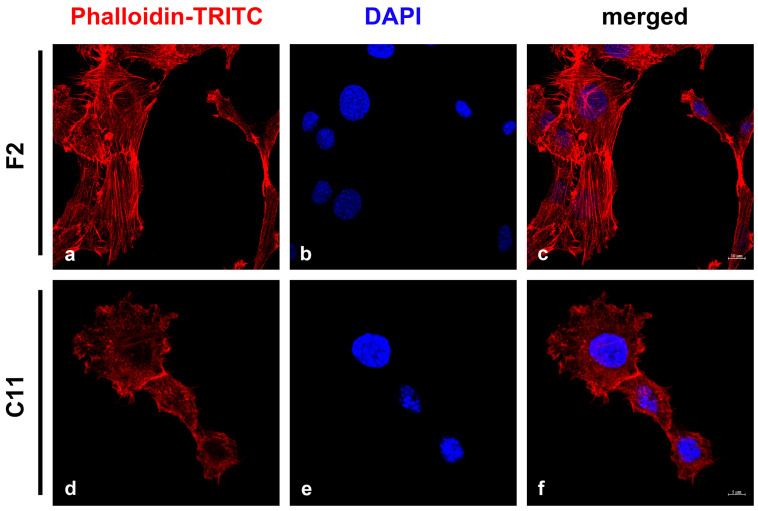
Detection of stress fibers in NIH3T3 sub-cell lines F2 and C11. The microfilament system was detected by Phalloidin-TRITC (**a**,**c**,**d**,**f**; red). Two patterns of labeling were observed: either long and parallel stress fibers (**a**) or diffuse labeling (**d**). The percentage of cells with stress fibers was calculated by manual counting. Nuclei were counterstained with DAPI (**b**,**c**,**e**,**f**; blue). Bars: F2: 10 μm, C11: 5 μm.

The obvious differences in size and morphology of the F2 and C11 sub-cell lines indicated functional heterogeneity and were reminiscent of fibroblasts and myofibroblasts. Therefore, we investigated whether the two sub-cell lines represented fibroblasts or myofibroblasts, which can be distinguished by differences in stress-fiber formation [6,36]. Stress fibers were decorated with Phalloidin-TRITC, a bicyclic peptide that binds to F-actin polymers [37]. The auto-fluorescence of the TRITC-labeled Phalloidin allowed the identification of stress fibers and the counting and categorization of cells into those harboring long and parallel stress fibers (first category) and those with short actin fibers or without any (second category) (Figure 3). We observed that 78% of F2 cells harbored typical stress fibers (333 cells of the first category/429 total counted cells), while only 33% of C11 cells contained stress fibers (160 cells of the first category/491 total counted cells). These observations suggested that both cell lines might be mixtures of myofibroblasts and fibroblasts with F2 cells consisting mainly of myofibroblasts and C11 cells, mostly fibroblasts.

**Figure 4:**
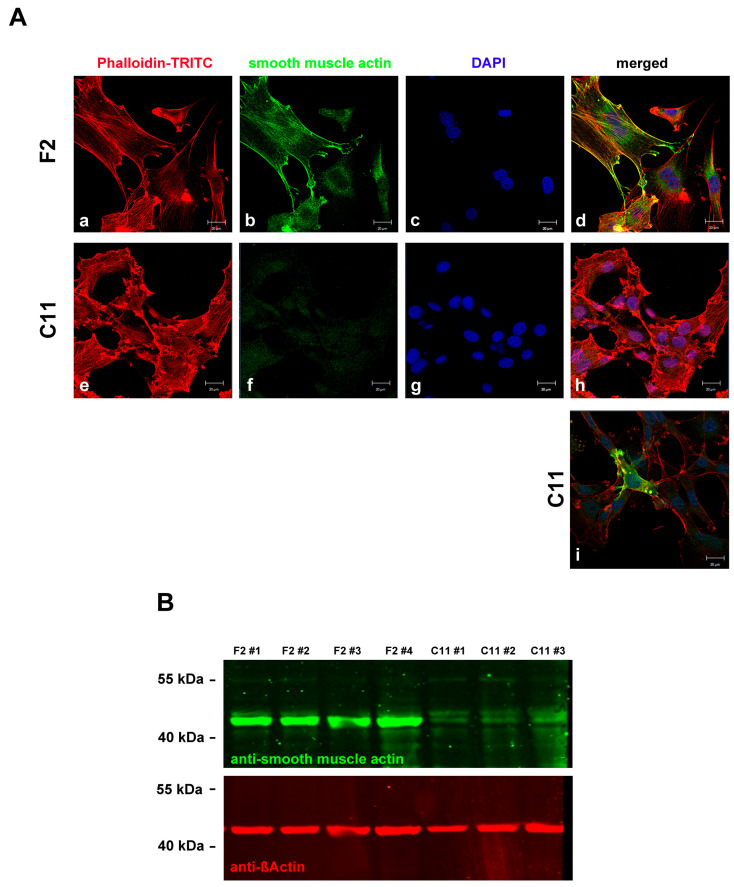
F2 cells are myofibroblasts. The expression of α-smooth muscle actin showed that F2 cells are myofibroblasts. (**A**) Detection of α-smooth muscle actin (**b**,**f**; green) and actin (**a**,**e**; red) in sub-cell lines F2 (**a**–**d**) and C11 (**e**–**i**). Nuclear staining with DAPI (**c**,**g**; blue) and merged images (**d**,**h**). Bars: 20 µm. All pictures were taken in identical settings. Only a few cells in the C11 sub-cell line expressed α-smooth muscle actin (**i**). (**B**) Quantitative Western blot showing increased expression of α-smooth muscle actin in the F2 sub-cell line. Four and three biological replicates (#1 to #4) were used for the F2 and C11 sub-cell lines, respectively. F2#1 to F2#3, and C11#1 and C11#2 were cultivated in serum-starvation medium, while F2#4 and C11#3 were cultivated in standard medium with 10% FCS. The same blot was incubated with antibodies against ß-Actin (red) and α-smooth muscle actin (green).

To demonstrate the diversity of F2 and C11 cells and their belongings to either myofibroblasts or fibroblasts, respectively, the cells were decorated for α-smooth muscle actin (Figure 4A). Almost all F2 cells expressed SMA, whereas in most C11 cells, SMA could not be detected (Figure 4A(a–d) versus Figure 4A(e–h)). However, in the majority of SMA-negative C11 cells, a few cells were found to express SMA (Figure 4A(i)). The differential expression of SMA in F2 and C11 cells persisted for at least 8-weeks of permanent cultivation, which was proven by Western blot analyses using protein lysates of proliferating and serum-starved cells (Figure 4B). Whereas SMA was strongly expressed in the F2 cells, only weak expression was found in the C11 cells. No effect of culture conditions on SMA expression was observed, as SMA expression did not change when cultured in either a standard medium or a serum-starvation medium.

### 3.3. Ciliation Differs in Sub-Cell Lines

The ability of the sub-cell lines to form primary cilia was examined immediately after their establishment. As usual, the same protocol was used to count cilia by seeding all sub-cell lines at a density of 2.5 × 10^5^ cells per 6-well and either culturing in standard medium containing 10% FCS (in short NM) for 24 h to support proliferation or further culturing in serum-starved medium containing only 0.5% FCS (in short SSM) to induce cell cycle arrest and formation of primary cilia for an additional 24 to 48 h. Primary cilia were decorated with both anti-acetylated α-tubulin and anti-ARL13B, both common ciliary marker proteins. Manual counting of the primary cilia revealed that the sub-cell lines differed greatly in ciliation, with the F2 cell line showing the highest percentage of cilia (81% ciliation in NM, 75% in SSM) and the C11 cell line having the lowest percentage of cilia (27% in NM, 46% in SSM) (Table 1). Interestingly, the proportion of cells with a primary cilium and the proportion of cells with stress fibers are similar (see above). Remarkably, serum-depleted medium stimulated cilia formation in C11 but not in F2 cells.

### 3.4. Different Proliferation Rates in the F2 and C11 Sub-Cell Lines

Our data indicated that the C11 sub-cell line appeared to be a fibroblast cell line, while the F2 sub-cell line more closely resembled myofibroblasts. Furthermore, the high percentage of ciliated cells in the F2 sub-cell line indicated a resting stage, in contrast to the C11 sub-cell line, which appeared to be predominantly in the proliferation stage when cultured in NM. This view is confirmed by our observation that serum deprivation stimulates cell cycle arrest and ciliation only in C11 cells.

**Figure 5:**
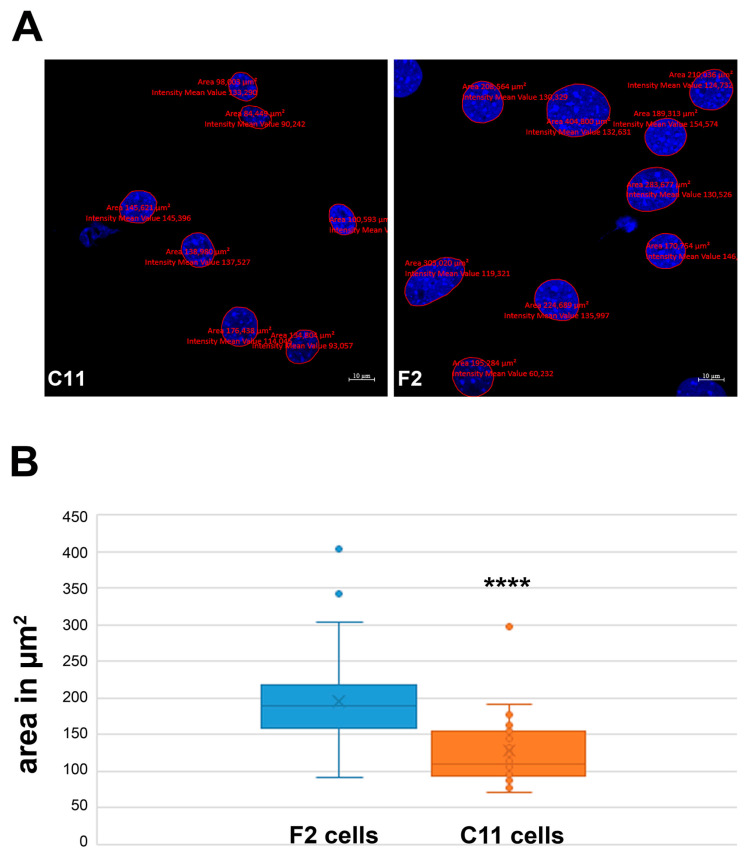
F2 and C11 sub-cell lines differ in nuclear size. (**A**) Nuclear size measurements in the C11 and F2 cells. Nuclear stain with DAPI (blue). Bars: 10 μm. (**B**) Nuclei of F2 cells are significantly larger than that of C11 cells with an average size of ~197 µm^2^ in F2 and of ~128 µm^2^ in C11 cells (*p* = 1.79156 × 10^−8^ ****). The cells were cultured in a standard medium containing 10% FCS. After fixation, the nuclei were visualized with DAPI, and the nuclear areas were measured using Zen blue software. For the measurements, nuclei from three biological replicates were randomly selected.

At first glance, we noticed an obvious difference in nuclear size between sub-cell lines F2 and C11, which would serve as a distinguishing feature between fibroblasts and larger myofibroblasts [8]. Measurements of the nuclear areas in both sub-cell lines revealed significant differences. On average, the nuclei of F2 cells were much bigger than the nuclei of C11 cells. Whereas the nuclei of F2 cells have a size of ~197 µm^2^, the nuclei of C11 cells have a reduced size of ~128 µm^2^ on average (Figure 5A,B). The two-sided Student’s *t*-test revealed a statistically significant difference (*p* = 1.79156 × 10^−8^ ****, two-sided, homoscedastic) (Table 2). Therefore, these results are consistent with our earlier data that the F2 cells seem to be mainly composed of myofibroblasts.

Considering the presumed correlation between cell size or nuclear size and the cell cycle, we examined the mitotic index and the cell cycle phases of the F2 and C11 sub-cell lines.

For the mitotic index, cells were seeded at a density of 2.5 × 10^5^ cells per well of a 6-well plate and cultivated in standard medium for 24 h followed by fixation and nuclear staining with DAPI. The mitotic indices were calculated by manually counting cells in the metaphase, as well as those that have finished mitosis but are still attached by cytoplasmic bridges (named here ‘double cells’) (Table 3). We observed that C11 cells had a higher mitotic index and more double cells than those found in the F2 cell line. These results indicate that C11 cells have a higher proliferation rate than F2 cells, confirming the differences in ciliation and also indicating that F2 cells are likely to be myofibroblasts and C11 cell fibroblasts, which is supported by the observation of a higher proliferation rate of fibroblasts than of myofibroblasts by [38].

**Figure 6:**
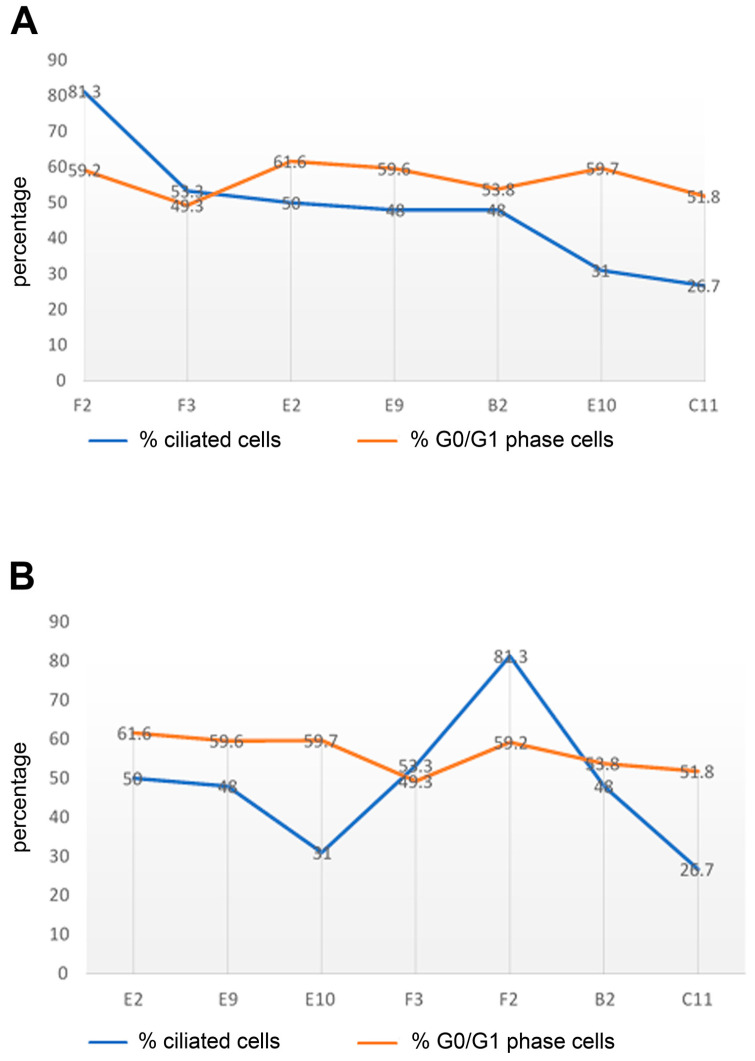
Correlation between ciliation, cells in the G0/G1-phase, and cell confluency. Indicated cell lines were grown in NM and processed for ciliary counting and FACS assays. (**A**) Comparison between the percentage of ciliated cells and cells in the G0/G1-phase. The F2 sub-cell line with the highest rate of ciliation (81.3%) also showed a high number of G0/G1-phase cells (59.2%), whereas the lowest ciliation rate in the C11 sub-cell line (26.7%) correlated with a low number of G0/G1-phase cells (51.8%). (**B**) The effect of cell confluency on ciliation and the cell cycle phase. The percentages of ciliation and G0/G1-phase cells were related to cell confluency, as estimated by visual inspection of all growth areas. Sub-cell lines were aligned from the most confluent on the left to the least confluent on the right. No correlation between ciliation and cell confluency was found. Nonetheless, comparing the extremes (E2 to C11 cells), a slight decrease in the percentage of G0/G1-phase cells was found when the cells were less confluent (C11 cells).

Furthermore, we analysed the percentage of cells in the G0/G1-phase by FACS assays of cycling cells. All cells were seeded at a density of 2.5 × 10^5^ cells per 5 mL flask and incubated for 3 days in NM. A comparison of the sub-cell lines in terms of the percentages of ciliated cells with cells that are in the G0/G1-phase, the phase of the cell cycle in which primary cilia are found, showed no obvious correlation (Figure 6A). We found that the F2 sub-cell line, which had the highest ciliation rate of 81.3%, had a high number of cells in the G0/G1-phase (59.2%), while the C11 sub-cell line, of which only 26.7% of cells had a primary cilium, had only 51.8% of cells in the G0/G1-phase. However, for the sub-cell lines with a proportion of ciliated cells of about 50% (F3 to B2), the percentage of cells in the G0/G1-phase ranged from 49.3% to 61.6%. Altogether, a weak correlation between the proportion of ciliated cells and cells in the G0/G1-phase was observed (Pearson correlation coefficient r = 0.477). To obtain reliable and comparable results in the FACS assays, identical cell counts for all sub-cell lines were initially seeded and cultivated. However, the proliferation rate seemed not to be identical for all sub-cell lines, as we observed different confluences when examining the whole areas of the cell culture dishes by light microscopy. As an additional indicator of cell proliferation, we inspected the color of the cell culture medium, which turned yellow when exhausted. To account for cell density and its effect on cell proliferation and ciliation, we arranged the sub-cell lines and their proportion of ciliated cells and cells in the G0/G1-phase according to the descending culture confluence, as observed by visual inspection, from the most confluent sub-cell lines (to the left) to the least confluent sub-cell lines (to the right) (Figure 6B). In this case, cell density and the percentage of cells in the G0/G1-phase appeared to correlate.

### 3.5. Ciliation and Proliferation of F2 and C11 Cells after Prolonged Cultivation

The cells were permanently cultivated in a standard medium by regular passaging. Approximately eight weeks after the initial establishment of the single-cell sub-lines, we observed a striking reduction in ciliation. When cultivated under proliferating conditions, i.e., standard medium with 10% FCS (in short normal medium, ‘NM’), only ~24% of F2 cells were ciliated (compared to ~81% when investigated shortly after the establishment of the sub-cell line), whereas C11 cells had only 8.5% primary cilia (compared to ~27% shortly after the establishment of the sub-cell line) (Table 4), which is a reduction to ~29% and ~32% in F2 and C11 cells, respectively, compared to their original number of primary cilia. Primary cilia, decorated with both ARL13b and acetylated α-tubulin, were manually counted in two biological replicates, each prepared within one week. FACS assays of proliferating cells revealed ~56.4% and 48.5% G0/G1-phase cells in the F2 and C11 sub-cell lines, respectively, which is an approximate reduction to 94–95% of the proportion of G0/G1-phase cells obtained in the first assays immediately after the establishment of sub-cell lines. However, in this case, FACS assays were done on three biological replicates each by seeding 400,000 cells per 5 mL flask followed by 24 h cultivation in NM, as opposed to the first FACS assay, in which only 2.5 × 10^5^ cells were seeded but cultivated for three days. Considering a duplication time of ~24 h, about twice the number of cells should be expected in the first FACS assay, which, most probable, might account for the higher proportion of cells in G0/G1-phase. Altogether, a reduced number of cells in the G0/G1-phase is therefore accompanied by a decrease in ciliation, and vice versa, but the correlation is only weak to moderate (Pearson correlation coefficient r = 0.44195006 for all probes together, including those of Figure 6).

**Figure 7:**
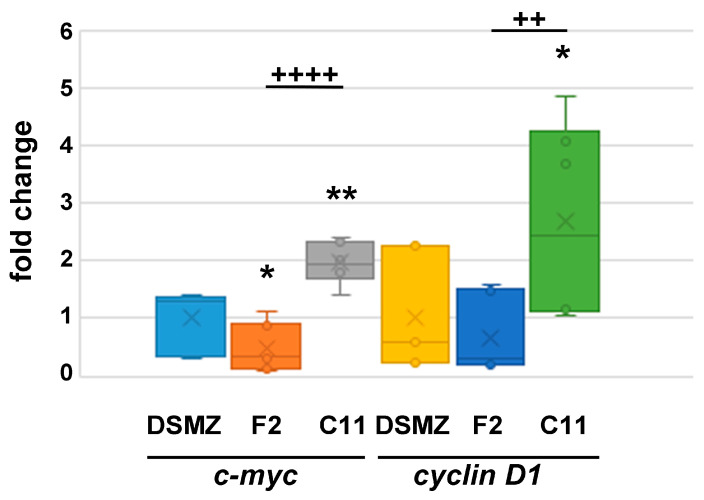
Quantitative expression of *c-myc* and *cyclin D1* in proliferating cells. The cells were cultivated under proliferating conditions. The relative expression was calculated by ΔΔCt using ‘DSMZ’ cells as the reference. NIH3T3 sub-cell line C11 showed increased expression of *c-myc* (*p* < 0.01 **) and *cyclin D1* (*p* < 0.05 *), whereas F2-cells showed a reduced expression of *c-myc* (*p* < 0.05 *). Compared to F2 cells, C11 cells have a significantly higher expression of *c-myc* (*p* < 0.0001 ^++++^) and *cyclin D1* (*p* < 0.01 ^++^).

To further confirm the different proliferation rates, we analyzed gene expression in proliferating cells cultured for 24 h in standard medium after reseeding using F2 and C11 cells grown permanently for about eight weeks. Compared to the original NIH3T3 ‘DSMZ’ cell line, we found reduced expression of *c-myc* in F2 cells (0.4749-fold, *p* = 0.02786072 *) and increased expression in C11 cells (1.9678-fold, *p* = 0.00168031 **). The change in expression of *cyclin D1* was not significant in F2 cells (0.6697-fold, *p* = 0.40637907) when compared to that of ‘DSMZ’ cells but significantly increased in C11 cells (2.6697-fold, *p* = 0.01751517 *) (Figure 7). Altogether, C11 cells have a significantly higher expression of *c-myc* (*p* = 5.44293 × 10^−6^ ****) and *cyclin D1* (*p* = 0.00710115 **) than F2 cells. We used 2 to 3 biological replicates for each cell line, giving a total of *n* qRT-PCR reactions: ‘DSMZ’ *n* = 9, F2 *n* = 9, C11 *n* = 6. The higher expression of *c-myc* and *cyclin D1* in C11 cells is consistent with their higher mitotic index figured out earlier, both indicating faster proliferation of C11 cells. Furthermore, the different proliferation rates are stably maintained for at least 8-weeks of permanent cultivation.

**Figure 8:**
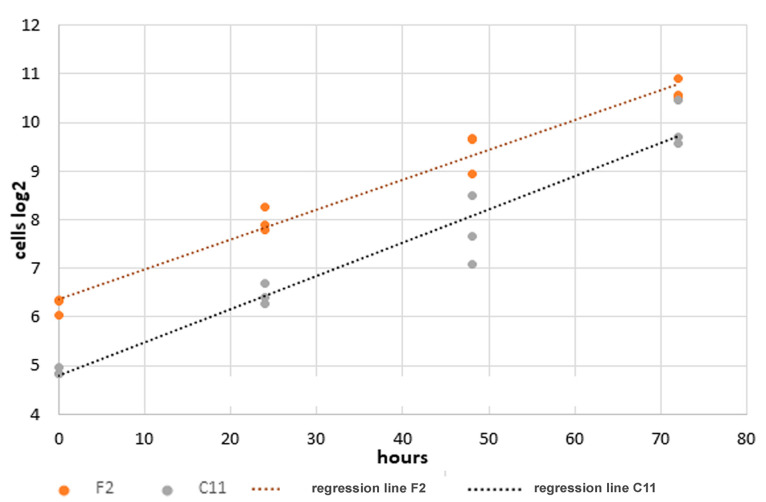
Increased growth rate of NIH3T3 sub-cell line C11. Identical cell numbers were seeded into 6-well plates and cultivated in standard medium with regular medium exchange at two-day intervals. Calculation of the doubling times is based on the binary logarithms of the cell counts using the inverted slope. Accordingly, the doubling time of the F2 cells was calculated as 16.34 h and that of the C11 cells as 14.63 h.

Finally, we examined the doubling time of the F2 and C11 cells after about eight months of permanent cultivation. The proliferation of F2 and C11 cells in standard medium was analyzed for 6-days, and their doubling time was calculated from the first 4-day interval when cells are in their exponential growth phase. The cells were initially seeded at constant numbers, and biological triplicates were analyzed at daily intervals. The nuclei were fixed and stained in situ, and 20 pictures were taken at random positions from each replicate. Nuclei were counted automatically, and the doubling time in hours was calculated from the inverted slope of the regression line obtained from the binary logarithms of the average cell counts at each time point. The doubling time of F2 cells was calculated as 16.34hrs whereas C11 cells had only a doubling time of 14.63 h (Figure 8). These results confirm the FACS assays, which showed that a higher proportion of F2 cells is in the G0/G1-phase than C11 cells, and are consistent with our initial observations that F2 cells generally grow slower and reach confluency later than C11 cells. Furthermore, ciliation is inversely correlated with cell proliferation.

To sum up, we have established sub-cell lines from single cells isolated from the commercially obtained mouse fibroblast cell line NIH3T3 (‘DSMZ’). Two of these cell lines, F2 and C11, representing the edges of the span in ciliation, differed in morphology and proliferation. The morphological signatures indicated that F2 cells were most likely myofibroblasts whereas C11 cells were most likely fibroblasts. The higher proliferation rate of C11 cells supported this classification, which was confirmed by Vaughan [38], who demonstrated a higher proliferation of fibroblasts than myofibroblasts. Both cell lines are stably maintained for at least 8 months of permanent cultivation, retaining their different proliferation rates and abilities to generate primary cilia. However, in both cell lines, we observed a decrease in the proportion of ciliated cells.

## 4. Discussion

Mouse embryonic fibroblasts (MEFs) are widely used in life science research. Notably, when treated with mitomycin to disrupt cell proliferation, they are used as feeder layers to support the propagation of embryonic stem cells. MEFs, like most other primary cell lines, have only a limited lifespan. In contrast, the mouse embryonic fibroblast cell line NIH3T3, which was generated by Todaro and Green in 1963, has become immortal through repeated transmission [25]. NIH3T3 cells have since been used in many research laboratories all over the world and have become one of the most popular cell lines in life science research (http://www.ncbi.nlm.nih.gov/pubmed, accessed on 1 June 2022). The NIH3T3 cell line was established from a Swiss mouse embryo of 17- to 19-days of gestation. The gestation length of mice was between 18 and 22 days. It is genetically determined and thus varies between different inbred strains [39]. However, the mouse embryos used for the generation of the NIH3T3 fibroblast cell line are fully developed and are available just shortly before birth. It is therefore expected that the cell line contains diverse cell types of the fibroblast lineage between those specific to their organ of residence [3,40]. On the other hand, due to their long-term propagation in culture, homogenization of the cell population may have occurred, with the slower proliferating fibroblasts outcompeted by the more proliferative ones. High-resolution cytogenetic characterization revealed that the cell line was relatively homogenous [41]. The NIH3T3 cell line has been propagated for approximately 60 years and is now commercially available by several different companies. Therefore, it is conceivable that a strain obtained by one company may not be identical to the same cell line obtained by another company. We have shown that the NIH3T3 cell line (obtained from the DSMZ in 2019) is heterogeneous. Using single-cell propagation, we generated two sub-cell lines, F2 and C11, which most likely represent a myofibroblast and a fibroblast line, respectively, according to the criteria of gene expression and proliferative capacity, as summarized in Table 5.

Myofibroblast-like sub-cell line F2 showed strong expression of SMA, which served as the predominant feature of myofibroblasts [7]. Fibroblasts have been identified by many studies as the source of myofibroblasts, but alternative origins have also been suggested, including endothelial cells, macrophages, and mesothelial cells. Thus, the origin of myofibroblasts is not fully clear [4,42]. During wound healing, fibroblasts are transformed into proto-myofibroblasts and myofibroblasts. Proto-myofibroblasts are defined as α-SMA-negative but stress-fiber positive transitional phenotype that may also reflect the reversibility of the myofibroblast phenotype [6,11,43]. However, the fate of the myofibroblasts is not properly known, as there is no expression of α-SMA in the residing cells in the wound. Thus, it has been proposed that myofibroblasts may either undergo apoptosis or return to the normal fibroblast phenotype without α-SMA expression. Interestingly, in recent years, heart myofibroblasts have been reported to morph into a new differentiated state called ‘matrifibrocytes’ after wound healing, and it was proposed that the former approaches were based only on α-SMA detection and loss of α-SMA expression would have been interpreted as apoptosis of myofibroblasts [44,45]. The activation of fibroblasts in wound healing depends on the upregulation of cell cycle proteins, p38-MAPK (mitogen-activated protein kinase), and ERK1/2 signalling pathways [46], reaching their maximum proliferation rate in 2–4 days [45]. Myofibroblasts then produce extracellular matrix (ECM; fibronectin (FN), collagen) components, giving the contractile ability to myofibroblasts and leading to the closure of the granulation tissue [47].

The F2 subline showed strong expression of SMA for at least 8 weeks of permanent cultivation. Additionally, F2 cells have a slower proliferation rate than the fibroblast-like sub-cell line C11. SMA-positive cells constitute a minor portion of the whole cell population of the original NIH3T3 cells obtained from the DSMZ, which might be caused by outcompeting this slower proliferating cell type. The fibroblast-like sub-cell line C11 is basically SMA-negative by immunofluorescence. However, as a minority of cells stained SMA-positive albeit the cell line originated from a single cell by clonal expansion, SMA-positive cells are most likely derived from SMA-negative, fibroblast-like cells by trans-differentiation. Trans-differentiation of fibroblasts into myofibroblasts seems to be a regular event caused by the reseeding of cells at low densities [8].

The myofibroblast-like subline F2 maintained a slower proliferation rate than the fibroblast-like subline C11 for at least eight months of permanent cultivation but persisted. Initially, it was suggested that all myofibroblasts undergo apoptosis after wound healing but recently their dedifferentiation back into fibroblasts or their trans-differentiation into another cell type, e.g., matrifibrocytes in the adult mammalian heart, has been proposed [45]. We observed no signs of apoptosis or a reduction in F2 cells, indicating their stable maintenance. The slower proliferation rate of the F2 cells is confirmed by their higher proportion of cells in the G0/G1-phase, which was demonstrated for the first eight weeks of cultivation. Furthermore, F2 cells showed lower expression of *c-myc* and *cyclin D1* compared to C11 cells.

c-Myc is a master regulator of genes involved in various cellular processes. It regulates cell proliferation by binding to E-boxes and acting as a transcription factor, and it also regulates translation and DNA replication [48]. The expression of c-Myc correlates strongly with cell proliferation, which is underlined by the observation that c-Myc-deficient fibroblasts show a remarkably prolonged doubling time [49,50]. The level of endogenous c-Myc protein is inversely proportional to cell density, such that low cell confluence is correlated with high levels of endogenous c-Myc protein [51]. In our case, the F2 and C11 cells were seeded with the same number of cells to obtain reliable and comparable gene expression results. The higher expression of *c-myc* together with *cyclin D1* in C11 cells therefore most likely reflects their increased proliferation rate.

Additionally, c-Myc mediates the suppression of growth arrest induced by the potent growth inhibitor TGF-β (transforming growth factor-ß) [52]. TGF-β induces the trans-differentiation of fibroblasts into myofibroblasts, which is manifested by the initial growth of a primary cilium followed by its loss when the myofibroblast state is finally reached [1,9,11]. Immediately after the establishment of single-cell colonies, we found that F2 cells are highly ciliated, having a proportion of cells with a primary cilium of ~81% when cultured in standard medium. No further increase in the proportion of ciliated cells could be induced when cultivated in serum-deprived medium. It is therefore obvious to interpret the high proportion of ciliated cells, the high expression of SMA, and the reduced proliferation rate as signs of myofibroblastic transformation. The remarkable reduction in the proportion of ciliated cells at a later time point could therefore be explained by the constitution of the final myofibroblast state.

Primary cilia are crucial organelles that transmit chemical and mechanical stimuli and regulate cell proliferation and differentiation [18,19]. The formation of primary cilia is tightly linked to the cell cycle. They are typically found during the G1- and G0-phases of the cell cycle and disassemble before mitosis [31,53]. There is increasing evidence that primary cilia are not generated simply as a consequence of the differentiated state of the cell, but that they function as structural checkpoints for cell cycle re-entry and progression [54]. Our data show that the proportion of ciliated cells moderately correlates with the proportion of cells in the G0/G1-phase of the cell cycle, thus indicating the interdependence of ciliation and cell cycle. We observed a reduction in the proportion of ciliated cells over time, not only for the F2 but also for the C11 cell line. The reason for this reduction in C11 cells is currently unknown but might be explained by a trans-differentiation into another unknown cell type.

To sum up, we established NIH3T3 sub-cell lines by clonal expansion that differ in proliferation and ciliation. These cell lines are valuable for more detailed studies of the molecular signatures that contribute to the formation of the primary cilium, as they exclude intercellular diversities present in the original NIH3T3 cell population.

## Figures and Tables

**Table 1:** Sub-cell lines differ in their ciliation capacities.

NIH3T3 Sub-Cell Lines	Ciliated Cells/Total Cells Counted (% of Ciliated Cells). Cells Were Cultivated in Standard Medium with 10% FCS (NM).	Ciliated Cells/Total Cells Counted (% of Ciliated Cells). Cells Were Cultivated in Serum Starved Medium (SSM).
F2	360/443 = 81.3%	399/533 = 75%
F3	244/458 = 53.3%	317/583 = 54%
E2	237/475 = 50%	
B2	214/446 = 48%	333/578 = 58%
E9	224/468 = 47.8%	
G3	186/468 = 39.7%	245/487 = 50%
E10	171/556 = 31%	
C11	127/475 = 26.7%	235/508 = 46%

*Note*. Sub-cell lines were grown in NM or SSM, and ciliation was quantified by immunological decoration of primary cilia and manual counting using the LSM (63×).

**Table 2:** Nuclear size differences in sub-cell lines.

Cell Line	AV ^1^	SD ^2^	N ^3^	*t*-Test
F2	196.60	53.0001	55	
C11	127.98	52.1368	38	1.79156 × 10^−8^ ****

*Note.*^1^ average, ^2^ standard deviation, ^3^ number. *p* < 0.0001 ****, two-sided, homoscedastic.

**Table 3:** Mitotic indexes of the sub-cell lines.

	F2	C11
Mitotic index	4/498 = 0.8% ~12 cells in telophase ^2^	25/468 = 3.6%
Double cells ^1^:	29/495 = 5.9%	57/506 = 11%

*Note.*^1^ mitosis is finished, but duplicated cells are still connected by cytoplasmic bridges indicative of a previous mitosis ^2^ cells are in their final mitotic phase and were not included in the calculation. The cells were visualized by LSM and counted (mitotic or double cells/total cells). Triplicate experiments.

**Table 4:** Decrease in the proportion of ciliated cells after eight weeks of permanent cultivation.

	%	F2	F3	E2	B2	E9	E10	C11
Immediately after the establishment of sub-cell lines	Ciliation	81.3%	53.3%	50%	48%	48.8%	31%%	26.7%
	G0/G1 phase	59.2%	49.3%	61.6%	53.8%	59.6%	59.7%	51.8%
After ~8 weeks of cultivation	Ciliation	23.6% (90/364/, 140/610)						8.5% (34/508/, 47/447)
	G0/G1 phase	56.4% (55.1%, 53.3%, 60.7%)						48.5% (48.2%,48%, 49.4%)

*Note*. Cilia were decorated with anti-acetylated α-tubulin and ARL13B, visualized by LSM, and were manually counted. The ratio illustrates the ciliated cells by the total number of counted cells. Cilia were mainly counted by ARL13B and checked by acetylated α-tubulin. In the case of difference, the average was considered. Cells were seeded at 2.5 × 10^5^ cells in a 6-well plate and cultivated for 24 h in NM. For the FACS assays, the cells were cultivated in NM. % ciliation in blue, % G0/G1-phase in green.

**Table 5:** Summary of morphological and molecular characteristics of the F2 and C11 sub-cell lines.

	F2	C11
morphology	cubic-shaped	spindle-shaped
% cells with stress-fibers (by Phalloidin-TRITC-staining)	78%	33%
α-SMA staining (immune-fluorescence)	+(almost all cells)	most cells negative, only few cells+
α-SMA (Western blot)	strong expression	weak expression
ciliation immediately after establishment of sub-cell lines	81% (in NM),	27% (in NM),
	75% (in SSM)	46% (in SSM)
nuclear size	~197 µm^2^	~128 µm^2^
mitotic index (% cells in metaphase)	0.80%	3.60%
% cells in G0/G1-phase(FACS assay of cycling cells)	59.20%	51.80%
ciliation in NM (after ~8-weeks of permanent cultivation)	24%	8.50%
% cells in G0/G1-phase (FACS assay of cycling cells after ~8-weeks of permanent cultivation)	56%	48.50%
*c-myc* expression (qRT-PCR; compared to that of F2 cells)		↑
*cyclin D1* expression (qRT-PCR; compared to that of F2 cells)		↑
doubling time in hours after ~8-months of permanent cultivation	16.34 h	14.63 h

Abbreviations: α-SMA = α-smooth muscle actin; NM = standard medium with 10% FCS; SSM = serum-deprived medium containing 0.5% FCS; qRT-PCR = quantitative reverse transcribed polymerase chain reaction; + positive staining; ↑increased expression. Different colours are used for better readability.
